# Tyro3 deletion is protective in experimental autoimmune encephalomyelitis

**DOI:** 10.1111/imcb.70054

**Published:** 2025-08-08

**Authors:** Michele D Binder, Mohammad Asadian, Darnell Leepel, Gerry ZM Ma, Andrea Aprico, Liz Barreto‐Arce, Trevor J Kilpatrick, Sarrabeth Stone

**Affiliations:** ^1^ The Florey Institute of Neuroscience and Mental Health Parkville VIC Australia; ^2^ Present address: Department of Microbiology and Immunology Peter Doherty Institute for Infection and Immunity, University of Melbourne Parkville VIC Australia

**Keywords:** Gas6, interleukins, TAM receptors, type 2 immunity

## Abstract

Multiple sclerosis is a complex neurological disorder, involving both the adaptive and innate immune systems as well as the CNS. The interaction between these systems is complex, and as such, there is the potential for MS therapies to have conflicting effects in different tissues. It is therefore critical that in addition to tissue‐specific studies, system‐wide effects of potential therapeutic pathways are explored. The circulating protein Gas6 is a promising therapy to promote remyelination in people with multiple sclerosis. Gas6 is a ligand for the TAM family of receptor protein tyrosine kinases that are widely expressed in the immune system and in the CNS, highlighting the potential for multi‐system effects as a result of Gas6 treatment. In this study, we demonstrate that global genetic deletion of either Gas6 or the Gas6 receptor Tyro3 results in reduced disease severity following induction of experimental immune encephalomyelitis in mice. The reduction in severity was accompanied by increased expression of both IL‐4 and IL‐17A in Tyro3 KO mice lymph node tissue and decreased expression of both cytokines in spinal cord tissues. IL‐4 is a cytokine known to be protective in inflammatory demyelination in mice. Conversely, the cytokine IL‐17A is known to be pathological. The overall shift to reduced disease severity highlights the multi‐faceted role of TAM receptor signaling in inflammatory demyelination.

## Graphical abstract

Multiple sclerosis pathogenesis involves the complex interplay between the peripheral immune response and the central nervous system. Gas6 is a promising therapy to promote remyelination; however, Gas6 and its receptors (the TAM receptor family) are widely expressed in both immune cells and the central nervous system. Our study demonstrates that global genetic deletion of either Gas6 or the Gas6 receptor Tyro3 results in reduced disease severity following induction of experimental immune encephalomyelitis in mice, which was accompanied by altered T cell cytokine expression.

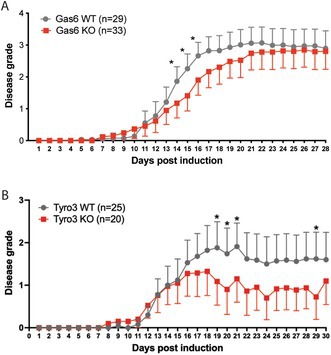

## INTRODUCTION

Multiple sclerosis (MS) is a chronic neurological disease of likely autoimmune origin. A plethora of immunotherapies can treat the inflammation and clinical activity of the early stages of MS. However, no therapy adequately treats the subsequent progressive phase of MS, which is characterized by ongoing demyelination, neurodegeneration, and inexorable functional decline.^1^ While axonal dysfunction and degeneration are the most direct causes of disease progression in MS, these deficits are a consequence of inflammatory demyelination. Inadequate remyelination compromises neural function and increases the susceptibility of axons to degenerate.^2^ One approach to treat progressive MS is to enhance myelin repair, thereby promoting functional recovery and limiting the propensity for otherwise demyelinated axons to degenerate.

The circulating protein Gas6 is a promising therapy for promotion of remyelination. The loss of Gas6 results in a worsened outcome following demyelination induced by the toxin cuprizone,^3^ and delays remyelination.^4^ Exogenous Gas6 administered directly to the CNS of mice improved functional outcome in an inflammatory model of MS (experimental autoimmune encephalomyelitis; EAE)^5^ as well as improved repair following cuprizone‐induced demyelination.^6^ Gas6, and the related Protein S (Pros1) are ligands for the TAM family of receptor tyrosine kinases, which is comprised of Tyro3, Axl, and Mertk.^7^ Gas6 is the only endogenous pan‐TAM receptor agonist, activating all three receptors.^8^ The three TAM receptors are expressed in the CNS but with distinct cell specificity: Tyro3 is expressed by oligodendrocytes and neurons while Mertk and Axl are expressed by microglia. We have recently demonstrated that promotion of remyelination by exogenously delivered Gas6 following cuprizone‐induced demyelination is at least partially dependent upon Tyro3 in oligodendrocytes.^9^ These studies provide strong evidence that Gas6 is an excellent pro‐remyelinating therapeutic candidate.

However, the expression of the TAM receptors is not limited to cells of the CNS but they are also widely expressed on cells of the innate immune system, including monocytes, dendritic cells, and macrophages.^10^, ^11^, ^12^ The TAM receptors are critical regulators of the immune response controlling the nature and magnitude of inflammation following pathological challenge. In the absence of all three receptors, TAM triple KO mice develop a broad‐spectrum autoimmune disorder.^12^ The TAM triple KO evidenced hyper‐proliferation of B and T cells secondary to loss of TAM receptors and their regulatory impact on macrophages and dendritic cells.^12^ More recently, subtleties in the regulation of TAM receptors have been identified, including TAM receptor expression on T cells as a component of regulation of T cell activation.^13^

As GAS6‐based therapies move toward clinical application, it is critical to have a complete understanding of the effect of TAM receptor activation upon inflammatory demyelination. Prior studies have identified that in EAE, disease course is worsened in the absence of Axl, accompanied by an increase in inflammatory cytokines in the spinal cord of Axl KO mice.^14^ Similarly, the loss of Gas6 worsened the course of EAE in mice and was associated with altered cytokine responses.^15^ Although these studies provide an important baseline for understanding the role of TAM receptors in inflammatory demyelination, information regarding expression of TAM ligands and receptors on circulating immune cell populations during the course of EAE remains limited. The role of Tyro3, in particular its influence upon immune cell activation and disease severity in the context of EAE, also remains unclear.

We therefore investigated the expression of TAM receptors and their ligands on circulating innate and adaptive immune cell populations during EAE in mice. Among the fluxes in expression we identified that expression of Tyro3 was upregulated in the T cell and dendritic cell populations over the course of EAE, while Gas6 was concomitantly upregulated in the B‐cell population. We subsequently induced EAE in Gas6 KO and Tyro3 KO mice. Contrary to our expectations, we found that deletion of either Gas6 or Tyro3 resulted in an improved disease course following induction of EAE. This improvement was not accompanied by changes to the number of lesions in the spinal cord, but was associated with an altered cytokine profile in the lymph nodes and spinal cords of Tyro3 KO mice.

## RESULTS

### TAM receptors and ligand expression is modulated during EAE

Interrogation of how TAM signaling modulates inflammatory demyelination requires a pre‐existing understanding of how this insult influences expression of TAM family members within both the CNS and circulating immune cells. To study this, EAE was induced in wild‐type C57BL/6 mice utilizing the autoantigen, MOG_35–55_, and immune cells purified and TAM gene expression assessed using quantitative PCR.

**Figure 1:**
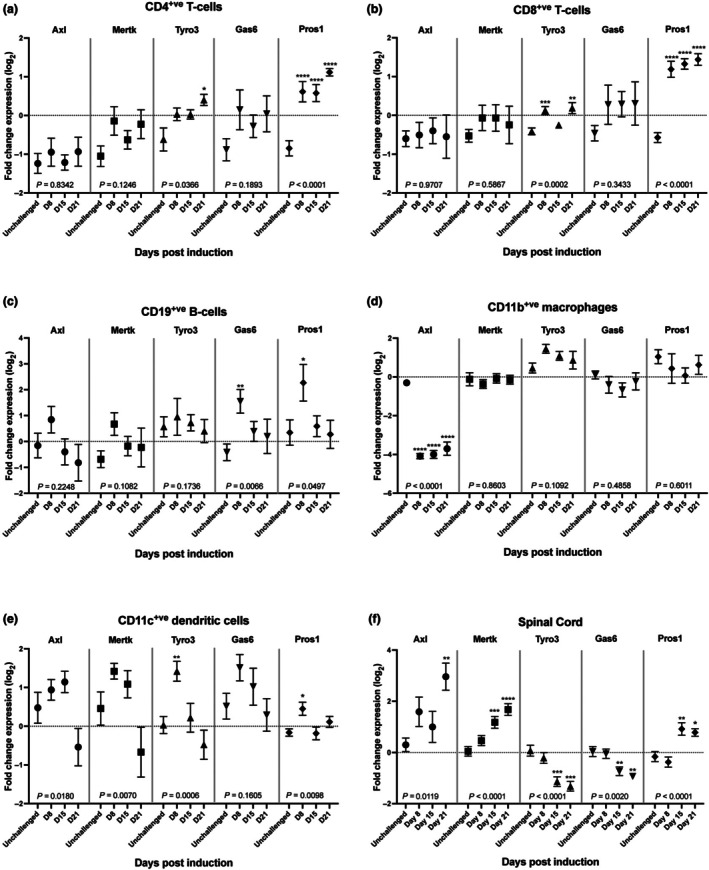
TAM receptor and ligand expression is altered in multiple immune cell types and in the CNS during the course of EAE. C57Bl/6 mice (*n* = 20 unchallenged; *n* = 15 D8; *n* = 14 D15; *n* = 7 D21, these mice formed a single cohort for PCR analysis, PCRs were performed once) induced with EAE were collected at 8, 15, and 21 days post‐induction, with unchallenged mice collected to establish baseline expression. Gene expression in immune cells purified from the spleen and in whole spinal cord tissue was assessed using qPCR. **(a, b)** The expression of both Pros1 and Tyro3 was significantly increased during EAE progression in both CD4^+^ and CD8^+^ T cells, with Pros1 showing a marked increase at all 3 time points, while a more moderate increase was observed for Tyro3. **(c)** The expression of both ligands was transiently increased in CD19^+^ B cells 8 days post‐induction, with no changes observed in receptor expression. **(d)** The expression of Axl was strongly downregulated in CD11b^+^ macrophages at all time points. **(e)** Axl, Mertk, Tyro3, and Pros1 were all significantly upregulated in CD11c^+^ cells early in the course of EAE, before returning to baseline levels by day 21. **(f)** In the spinal cord, the expression of Axl, Mertk, and Pros1 was increased over the course of EAE, peaking at 21 days post‐induction. Conversely, Tyro3 and Gas6 expression was significantly reduced over the same time period. Significant differences between the means were determined using one‐way ANOVA, followed by Dunnett's multiple comparison test. One‐way ANOVA results are indicated at the bottom of the graph, with significant *post‐hoc* tests above individual points. *P < 0.05, **P < 0.01, ***P < 0.001, ****P < 0.0001.

The expression of Tyro3 and Pros1 was modulated in splenic T cells. In the CD4^+^ T cell population, the expression of *Tyro3* was increased ~1.0 fold in EAE mice relative to unchallenged controls at 21 days post‐induction (d.p.i.) (Figure 1a). In the CD8^+^ T cell population, the expression of *Tyro3* which was modestly increased at 8 and 21 d.p.i. (Figure 1b). *Pros1* was strongly (~2 fold) and persistently elevated between days 8 and 21 in both the CD4^+^ and CD8^+^ T cell populations (Figure 1a, b). No significant changes in expression were detected in T cells for either *Axl, Mertk*, or *Gas6* (Figure 1a, b).

There was no significant variation in the expression profiles of any of the TAM receptor genes in B cells during the course of EAE. Transient but robust (twofold) increases in expression profiles of the genes encoding both TAM ligands were observed in the B‐cell population at 8 d.p.i. (Figure 1c).

Among CD11b^+^ leucocytes, there were no significant changes in the expression of any TAM family gene members, except for persistent downregulation of Axl (~4.0 fold at all timepoints; Figure 1d). On the other hand, the expression profiles of all the receptor genes and of Pros1 were altered in CD11c^+^ cells with *Tyro3* (1.5 fold) and *Pros1* (0.5 fold) both transiently increased at 8 d.p.i. (Figure 1e) with more persistent elevation of both *Axl* and *Mertk* (~1.0 fold increases for both genes at 15 d.p.i.).

Within the spinal cord, two complementary expression patterns were observed. There was a gradated increase in the expression of *Axl, Mertk*, and *Pros1* as the disease progressed (respective fold increases of 2.7, 1.6, and 0.95 at 21 d.p.i.), while downregulation of *Tyro3* and *Gas6* gene expression was identified, commencing at 15 days d.p.i. and persisting at 21 d.p.i. (−1.4 and − 0.97 fold at 21 d.p.i., respectively) (Figure 1f).

### Gas6 and Tyro3 worsen the course of EAE in mice

Having identified that the expression of the gene encoding Tyro3 was increased in both the T cell and dendritic cell populations during the course of EAE, we decided to assess the influence of its deletion upon disease severity and to compare and contrast this phenotype with that induced by the deletion of Gas6. Deletion of Tyro3 receptor has previously been demonstrated to worsen outcome in the CNS‐specific model of demyelination induced by the toxin cuprizone,^16^ but its role in inflammatory demyelination has not been investigated. We therefore induced EAE in both Gas6 KO mice and Tyro3 KO mice, as well as littermate WT mice using MOG_35–55_.

**Figure 2:**
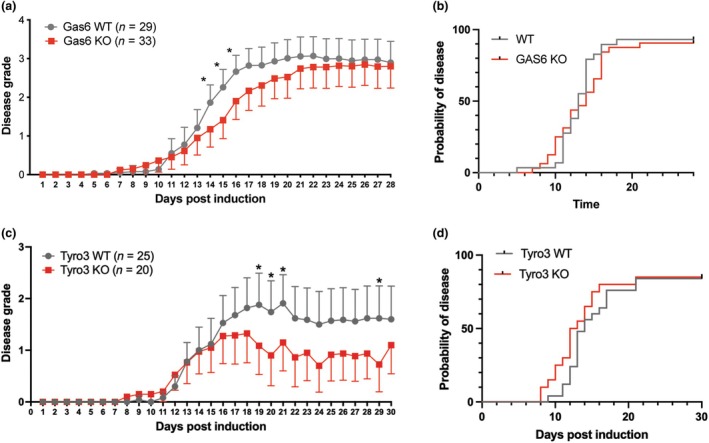
The loss of Gas6 and Tyro3 results in reduced disease during EAE. EAE was induced in Gas6 KO and WT mice (*n* = 29 Gas6 WT; *n* = 33 Gas6 KO, n represents pooled data from three individual cohorts) and Tyro3 KO and WT mice (*n* = 25 Tyro3 WT or 20 Tyro3 KO, n represents pooled data from three individual cohorts) using MOG_35–55_. **(a)** Mean disease grade was significantly reduced in the absence of Gas6 at Days 14–16 days post‐induction. **(b)** Disease incidence was not altered in Gas6 KO mice. **(c)** Mean disease grade was reduced in Tyro3 KO mice from 19 days following induction, reaching significance at Days 19 and 20 and Day 29. **(d)** Disease incidence was not altered in the absence of Tyro3. **(a, c)** Values represent mean ± s.d. Statistical differences in mean clinical grades were assessed using the Mann–Whitney *U*‐test. *P < 0.05. **(b, d)** Statistical differences in EAE incidence was assessed using the Gehan–Breslow–Wilcoxon method. *P < 0.05.

The loss of Gas6 unexpectedly resulted in a significant reduction in disease grade from 14 d.p.i., although the difference between groups was not maintained for the duration of the experiment (Figure 2a). The difference in disease course was not associated with a difference in disease incidence, mortality or day of onset (Figure 2b; Table 1).

**Table 1:** Characteristics of EAE in Tyro3 KO and WT mice.

Strain	Incidence	Mortality	Maximum disease grade	Average day of onset (days)
GAS6 WT	93% (*n* = 29)	55% (*n* = 29)	2.875 (*n* = 12)	13 (*n* = 27)
GAS6 KO	88% (*n* = 33)	58% (*n* = 33)	2.375 (*n* = 14)	13 (*n* = 29)
***P*‐value of difference**	**0.6757**	**> 0.9999**	**0.1133**	**0.8473**
TYRO3 WT	84% (*n* = 25)	20% (*n* = 25)	2.25 (*n* = 20)	13 (*n* = 21)
TYRO3 KO	85% (*n* = 20)	4% (*n* = 20)	1.75 (*n* = 19)	12 (*n* = 17)
***P*‐value of difference**	**> 0.9999**	**0.1552**	**0.5026**	**0.0964**

The bold text delineates the Gas6 and Tyro3 sections of the table; that is, bold values are the *P* values for the data in the two non‐bold rows above.

Similar to our observations in Gas6 KO mice, the loss of Tyro3 resulted in a reduction in disease grade from 17 days post‐induction (d.p.i.) until the conclusion of the experiment at 30 d.p.i. (Figure 2c). The improvement in disease grade was not the result of reduced disease incidence or mortality (Figure 2d; Table 1). Conversely, the loss of Tyro3 resulted in a trend toward an earlier disease onset (median 1 day) (Table 1). Overall, these data suggest that the Gas6‐Tyro3 signaling axis worsens disease course in MOG_35–55_ EAE in mice.

### Loss of Tyro3 is associated with altered cytokine profiles during the effector phase of EAE

**Figure 3:**
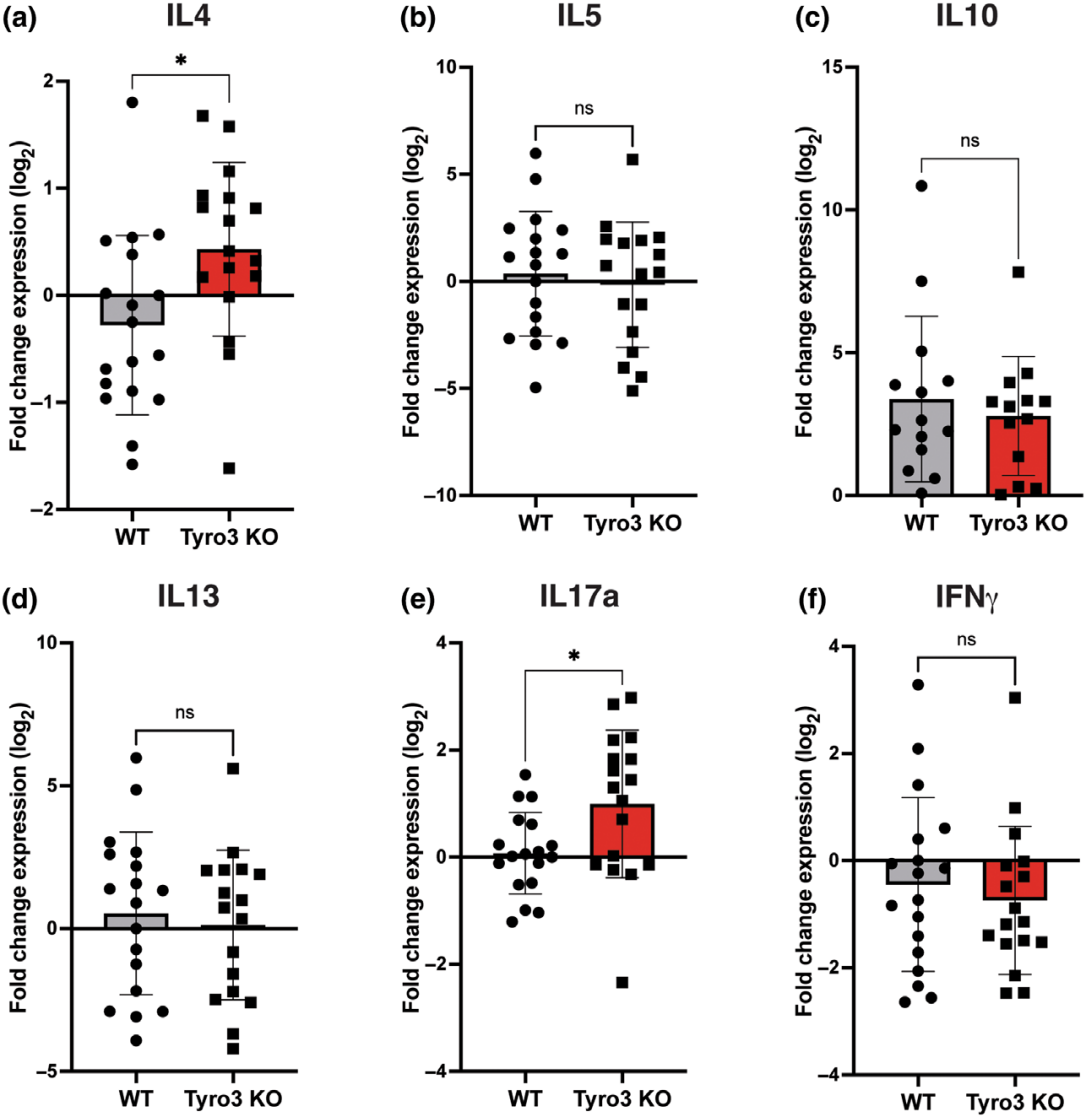
Cytokine expression is altered in the lymph nodes of Tyro3 KO mice in the early effector stage (16 d.p.i.) of EAE. EAE was induced in WT and Tyro3 KO mice (*n* = 17 or 18 per genotype, these mice formed a single cohort for PCR analysis, PCRs were performed once) using MOG_35–55_. The mRNA level of critical cytokines in total inguinal lymph nodes was assessed using qPCR. **(a)** The expression of *IL‐4* mRNA was significantly higher in the lymph nodes of Tyro3 KO mice compared with WT. No significant difference in expression was observed between Tyro3 KO and WT mice for *IL‐5* **(b)**, *IL‐10* **(c)**, or *IL‐13* **(d)**. **(e)** The expression of *IL‐17a* was significantly higher in Tyro3 KO mice compared with WT. **(f)** No significant difference in the expression of IFN‐γ between Tyro3 KO and WT mice was observed. Data are presented as mean ± s.d.; points represent individual mice. Statistical differences in gene expression were determined using the Student's *t*‐test; *P < 0.05, n.s. not significant.

We used qPCR to investigate the molecular correlates of improved disease course in the absence of Tyro3. Differences in disease score can be due to alterations in the extent of immune invasion in the CNS or type (e.g. Th1 vs. Th2 predominant) of immune response; however, feedback loops caused by differing levels of tissue damage can confound results if data are collected late in the disease course. Therefore, to avoid these potentially confounding molecular changes related to disease severity, we induced EAE in a separate cohort of WT and Tyro3 KO mice and collected inguinal lymph nodes and spinal cords at 16 days post‐induction prior to any difference in mean disease severity (Figure 2c), mRNA was extracted from these tissues. We first examined the expression of cytokines with known roles in EAE and MS pathogenesis in the inguinal lymph nodes (Figure 3a–f). We observed a significant increase in the expression of the type 2 cytokine interleukin (IL)‐4 (Figure 3a). The expression of other type 2 cytokines IL‐5, and IL‐13, and the anti‐inflammatory cytokine IL‐10 were similar between WT and Tyro3 KO mice (Figure 3b–d). We also observed significantly increased expression of the cytokine IL‐17a (Figure 3e) but not interferon (IFN)‐γ (Figure 3f) in lymph nodes derived from Tyro3 KO mice.

**Figure 4:**
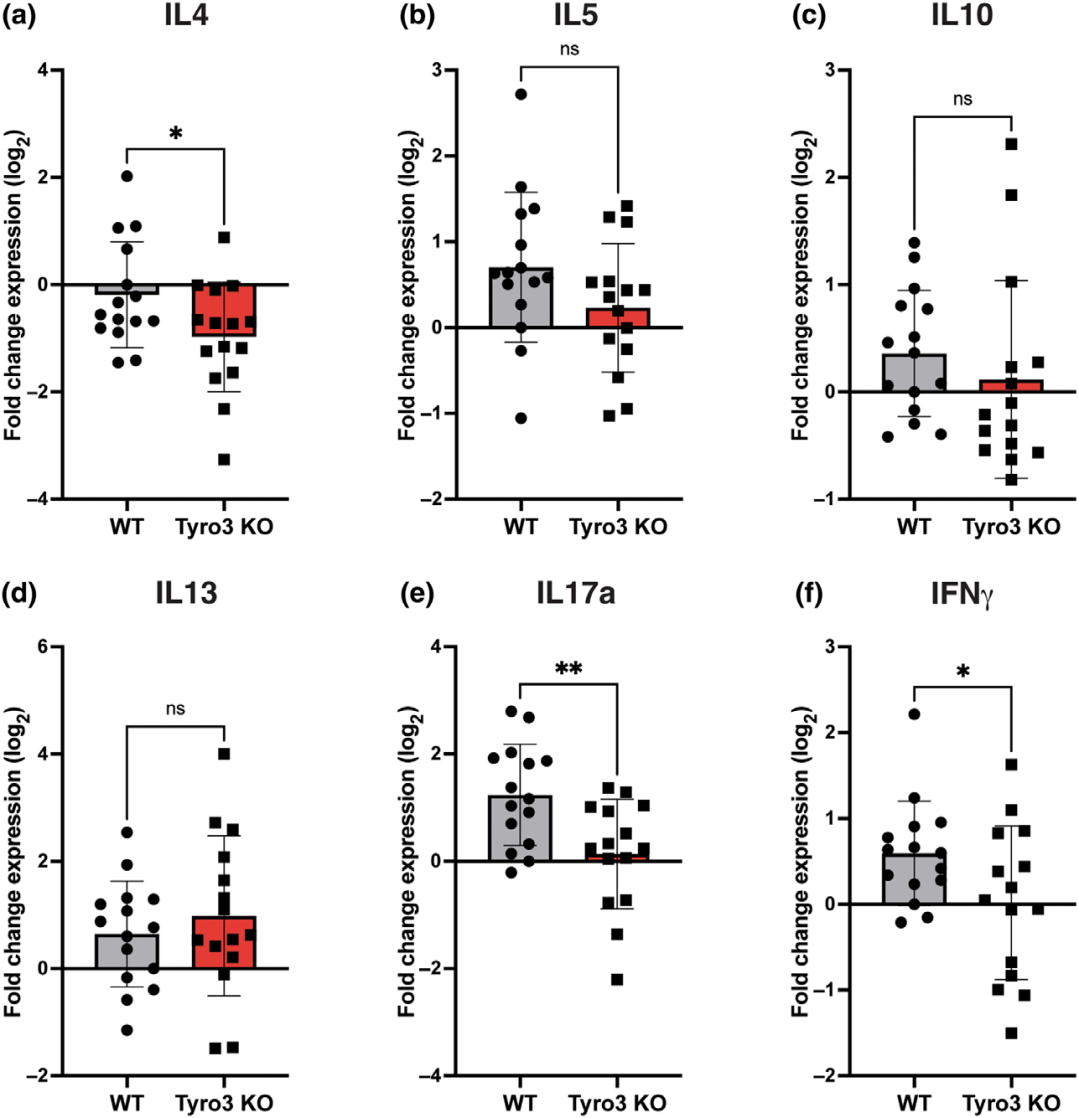
Cytokine expression is altered in the spinal cords of Tyro3 KO mice in the early effector stage (16 d.p.i.) of EAE. EAE was induced in WT and Tyro3 KO mice (*n* = 17 or 18 per genotype, these mice formed a single cohort for PCR analysis, PCRs were performed once) using MOG_35–55_. The mRNA level of critical cytokines in whole spinal cords was assessed using qPCR. **(a)** The expression of *IL‐4* mRNA was significantly lower in the spinal cords of Tyro3 KO mice compared with WT. No significant difference in expression was observed between Tyro3 KO and WT mice for *IL‐5* **(b)**, *IL‐10* **(c)**, or *IL‐13* **(d)**. **(e, f)** The expression of *IL‐17a* and IFN‐γ was significantly lower in Tyro3 KO mice compared with WT. Data are presented as mean ± s.d.; points represent individual mice. Statistical differences in gene expression were determined using the Student's *t‐*test, *P < 0.05, **P < 0.01, n.s. not significant.

In the spinal cord, in contrast to lymph nodes, loss of Tyro3 was associated with lower expression of IL‐4 compared with WT mice (Figure 4a). Consistent with our observations in lymph nodes, expression of IL‐5, IL‐10, and IL‐13 was similar between WT and Tyro3 KO mice (Figure 4b–d). However, the expression of both IL‐17a (Figure 4e) and IFN‐γ (Figure 4f) was significantly reduced in spinal cord derived from Tyro3 KO mice compared with WT mice. Taken together, these data demonstrate an altered pattern of cytokine gene expression that is occurring in a tissue‐specific and genotype‐dependent manner.

### Spinal cord lesions and microglial density in early EAE are not altered in the absence of Tyro3

**Figure 5:**
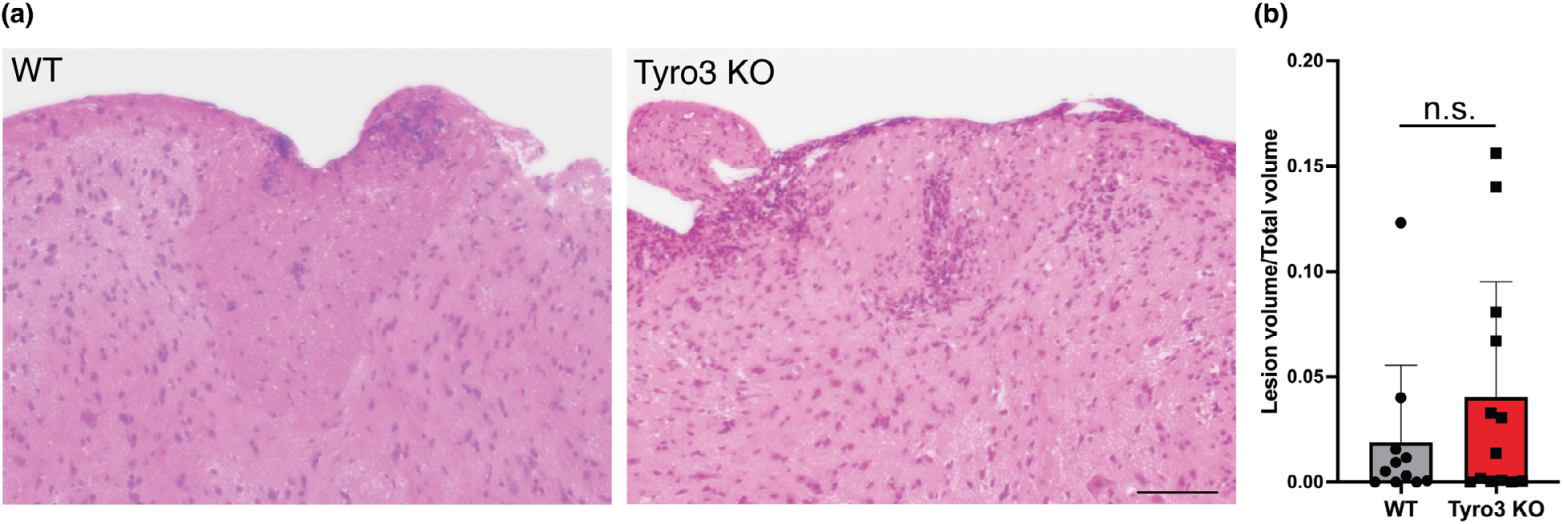
Spinal cord lesion density is not altered in Tyro3 KO mice in early EAE. EAE was induced in WT and Tyro3 KO mice (n = 17 or 18 per genotype, these mice formed a single cohort for lesion density analysis which was performed once) using MOG_35–55_, and spinal cords were harvested at 16 d.p.i. **(a)** Cells within the lumbar spinal cord region were visualized using H&E staining. Lesions within representative sections are outlined by white dotted lines. **(b)** Lesion density was not significantly different in Tyro3 KO mice compared with WT (*P* = 0.2822). Lesion density is expressed as the combined area of lesion(s) as a proportion of total spinal cord area, and error bars represent mean ± s.d.; points represent individual mice. Statistical differences were determined using the Student's *t*‐test. Scale bar 100 μm. n.s., not significant.

**Figure 6:**
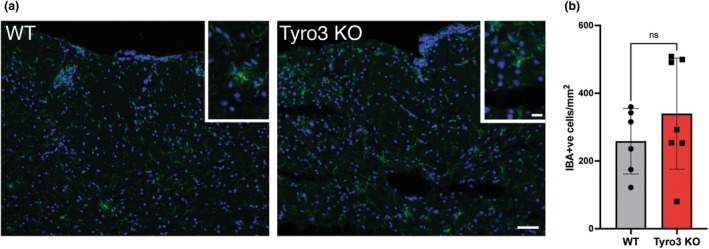
Density of IBA1^+^ microglia is not altered in Tyro3 KO mice in early EAE. EAE was induced in WT and Tyro3 KO mice (*n* = 6 or 7 per genotype, these mice formed a single cohort for IBA1^+^ density analysis which was performed once) using MOG_35–55_, and spinal cords were harvested at 16 d.p.i. **(a)** Microglia within the dorsal column of the spinal cord were visualized using anti‐IBA1 (green) immunofluorescence with all nuclei visualized using Hoescht (blue). Insets show IBA1^+^ microglia from within the same section. **(b)** The density of IBA1^+^ microglia was not significantly different in Tyro3 KO mice compared with WT. Error bars represent mean ± s.d.; points represent individual mice. Statistical differences were determined using the Student's *t‐*test. Scale bar 100 μm. n.s., not significant. Inset scale bar 25 μm.

We next examined whether the altered cytokine profile was associated with changes in the cellular profile of the inflammatory infiltrate in the CNS of the Tyro3 EAE mice. We first assessed the lesion load within the spinal cords of WT and Tyro3 KO mice using H&E staining (Figure 5a). The average area of lesions as a proportion of total spinal cord area was not significantly different between WT and Tyro3 KO mice (Figure 5b). In addition to lesion area, we also determined the density of IBA1^+^ microglia within the spinal cord (Figure 6a). We did not observe any significant difference in the density of microglia between WT and Tyro3 KO mice (Figure 6b), suggesting that the differences in cytokine expression in the CNS are unrelated to lesion area or density of microglia.

## DISCUSSION

In this study, we undertook a detailed examination of the expression of the TAM receptors and their ligands during experimentally induced inflammatory demyelination in mice, identifying altered expression of receptor and ligand mRNA in multiple immune cell types as well as in the spinal cord. Of note, we observed an increase in the expression of Tyro3 in both the dendritic cell and T cell populations in advance of the clinical manifestation of EAE. In contrast to our expectations, the induction of EAE in Tyro3 KO mice ameliorated disease. We identified that this improvement was associated with an increase in the expression of the type 2 cytokine IL‐4 and IL‐17A in the lymph nodes of Tyro3 KO mice and an accompanying decrease in the expression of IL‐4, IFNγ, and IL‐17a in the spinal cord. IL‐4 and IL‐17A have notable protective and pathological roles in EAE respectively, whereases the role of IFN‐γ is notably pleiotropic and likely context dependent.^17^, ^18^, ^19^ These data suggest that the reduction in EAE severity may be associated with a shift in T cell cytokine balance.

Signaling via the TAM receptors has been previously demonstrated to alter demyelination and remyelination in multiple pre‐clinical models of MS.^3^, ^4^, ^14^, ^16^, ^20^, ^21^, ^22^ However, there remained gaps in our understanding of the modulation of TAM receptors and ligands throughout the course of inflammatory demyelination, particularly in respect to their expression in circulating immune cells. Our examination of the expression of TAM receptors and ligands in adaptive and innate immune cells during the course of EAE revealed some general patterns. Firstly, and not unexpectedly, Pros1 expression was strongly increased in both CD4^+^ and CD8^+^ spleen‐derived T cell populations at all times assessed, encompassing both the induction and the effector phases of EAE. This finding is consistent with prior literature which has identified that Pros1 is induced in activated T cells and interacts with dendritic cells to limit the immune response to pathogens.^23^ From Day 15 following induction of EAE, Pros1 expression is increased in the spinal cord, likely due to the infiltration of T cells into the spinal cord which occurs from about Day 11 in this model.^24^

In contrast to Pros1, modulation of TAM receptor expression in spleen‐derived cells was generally restricted to innate immune cell populations, with the exception of Tyro3 which was significantly increased in both CD4^+^ and CD8^+^ T cell populations. In particular, expression of TAM receptors and ligands were significantly modulated in the splenic CD11c^+^ dendritic cell population. All three receptors were significantly increased during the early stages of EAE, but in general had returned to a baseline level by Day 21 following induction. As discussed above, it has previously been demonstrated that the interaction of Pros1 on activated T cells with Mertk on dendritic cells limits the immune response to pathogens, preventing the development of chronic inflammation.^23^ Additionally, Mertk has been demonstrated to be expressed by activated human T cells, where it has a pro‐proliferative function when activated by Pros1.^13^ It remains possible that Axl or Tyro3 have similar functions, but this has not been explicitly tested. In comparison, only Axl expression was altered in splenic CD11b^+^ macrophage with a significant decrease in expression at all EAE timepoints. Axl deficient mice experience exacerbated EAE; however, the cell specific role of Axl in inflammatory demyelination is not well understood.^14^ In macrophages a deficiency in Axl signaling has been associated with induction of pro‐inflammatory T cell responses and enhanced T cell‐induced M1‐like functions.^25^, ^26^ The reduced expression of Axl on splenic CD11b^+^ macrophages suggests they may have a role in promoting encephalitogenic T cell responses.

Expression of Tyro3 in the immune system was once thought to be limited to innate immune cells,^12^ and has not been described in non‐stimulated T cells. In this study, we observed a modest increase in the expression of Tyro3 in the CD4^+^ and CD8^+^ cell populations following EAE induction. A role for Tyro3 in T cells has previously been undescribed; however, these data raise the possibility that the influence of Tyro3 on inflammatory demyelination may extend beyond innate immune cells to T cells. Conversely, Tyro3 and Gas6 were significantly reduced in the spinal cord at days 15 and 21 following induction while Axl, Mertk, and Pros1 were increased, particularly at day 21 following induction. These timepoints correspond to early (day 15) and peak (day 21) infiltration of the spinal cord by the peripheral immune system. Tyro3 is highly expressed on neurons and oligodendroglia lineage cells and the reduction in Tyro3 expression may relate to loss of, or damage to, neurons, axons, and oligodendrocytes following immune invasion. At these time points substantial tissue damage occurs, the elevated levels of Axl and Mertk may be related to enhanced efferocytosis activity of resident cells (e.g. microglia), increased infiltration of immune cells, or a combination of the two. It is likely that reductions in Gas6, which is highly expressed in the CNS may be related to damage or loss of CNS cells, while increases in Pros1, which is highly expressed on T cells may be related to the infiltration of immune cells into the spinal cord.

In this study we identified a reduction of disease severity following induction of EAE in Tyro3 KO mice, notably we found no difference in the day on onset of incidence of EAE. This provides further support for the idea that Tyro3 is involved in the regulation of the immune response during inflammatory demyelination but does not suggest a change in priming or development of the immune response. Notably, we also observed a similar, although not as pronounced, reduction in disease severity in Gas6 KO. These data are surprising on a number of levels and highlights the complexity of TAM receptor signaling in inflammatory demyelination. In people with MS higher levels of serum PROS1 or GAS6 have been associated with reduced MS severity scores (MSSS) suggesting important roles for TAM signaling in disease pathogenesis.^27^, ^28^ Conversely in the cerebral spinal fluid (CSF) levels contradictory results have been found for GAS6 with one study finding no correlation between CSF GAS6 and MS severity scores and another finding increased GAS6 in more progressive disease.^27^, ^29^ TAM signaling pathways are further complicated by the presence of both membrane bound and soluble forms of the TAM receptors and it has been proposed that soluble receptors in MS lesions outcompete membrane bound TAM receptors for GAS6 leading to inefficient remyelination.^30^ This makes the roles of TAM signaling in MS difficult to determine based solely on expression of TAM receptors and ligands.

It has been well established that TAM receptor signaling in the CNS is protective in the context of demyelination and remyelination. The loss of any of the TAM receptors or Gas6 is known to worsen the outcome of cuprizone‐induced demyelination.^3^, ^16^, ^20^, ^21^, ^22^ Additionally, in three separate models of CNS demyelination (EAE, cuprizone‐mediated demyelination, and lysolecithin‐induced demyelination in organotypic slice culture), direct administration of exogenous Gas6 increased remyelination, and in EAE, reduced disease severity.^6^, ^9^, ^15^, ^31^ In the context of cuprizone‐mediated demyelination, the therapeutic effect of Gas6 is at least partly dependent on Tyro3^9^. Based on these findings, we similarly expected to observe a worsened outcome in EAE in Gas6 KO and Tyro3 KO mice. It has previously been shown that loss of Gas6 increases the severity of EAE,^15^ a result that stands in contrast to our own findings. It also remains unclear the extent to which each TAM receptor may contribute to the effect of Gas6 KO in EAE. One explanation for these contradictory findings could lie in the nature of EAE as a model, which can vary substantially between different laboratories dependent upon multiple factors. It is therefore reasonable to conclude that the role of Gas6 in inflammatory demyelination is context dependent and will require further study to unravel the underlying complexities. Nonetheless, our data identify that both Tyro3 and Gas6 can worsen the course of inflammatory demyelination in mice.

The explanation for these unexpected findings could lie with the role of Th2 (type 2) cytokines in EAE in mice. In general terms, type 2 cytokines such as IL‐4, IL‐5, and IL‐13 are considered to be anti‐inflammatory/protective. IL‐4 has been demonstrated to suppress Th1 immune responses, conferring protection against EAE.^32^, ^33^ Type 2 cytokines are associated with a host protective response to parasitic infections, such as helminths,^34^ but also drive allergic disease.^35^ It has previously been demonstrated that Tyro3 is a negative regulator of type 2 responses, suppressing key type 2 cytokines including IL‐4, IL‐5, and IL‐13, and Tyro3 KO mice show greater resistance to helminth infection but increased disease in a model of dust allergy.^36^ Suppression of the type 2 immune response was demonstrated in this prior study to be dependent upon Tyro3 expressed on dendritic cells and Pros1 expression in T cells.^36^ It is notable that the decrease in EAE severity observed in the absence of Tyro3 was substantially greater than that observed in Gas6 KO, suggesting a potential role for Pros1, the other Tyro3 ligand, in this phenotype. We found that in the early effector phase of EAE, prior to divergence of clinical symptoms, the expression of IL‐4 was significantly increased in the lymph nodes of Tyro3 KO mice compared with WT, supporting the hypothesis that the ameliorated disease observed in Tyro3 KO mice was associated with de‐repression of type 2 cytokines; although, as discussed below, we did not observe a concomitant repression of the type 1 cytokine IFN‐γ in the lymph node. Interestingly, the increase in type 2 cytokines appeared specific to IL‐4, as we did not observe any change in expression in IL‐5, IL‐10, or IL‐13. In contrast to the lymph nodes, we observed decreased expression of IL‐4 in the spinal cord of Tyro3 KO mice compared with WT prior to clinical disease. Although the mechanism underlying this disparity is unclear, it suggests that the critical interactions between dendritic cells and T cells which may ultimately result in Th2‐biased T cells occur outside the CNS. However, IL‐4 has multiple direct and indirect effects on the immune system, including on antigen presenting cells and on antibody production and class switching by B cells.^37^ For example, CD8^+^ T cells that interact with IL‐4 treated dendritic cells fail to acquire cytolytic functions.^38^ As both CD8^+^ T cells and antibodies have roles in EAE pathogenesis, it is possible that the increase in IL‐4, in the absence of an increase of other Th2‐associated cytokines, may instead be affecting the immune response directly to lower disease scores in EAE.^39^, ^40^

We did not observe any differences in the histopathological features of the infiltrate in the spinal cord of Tyro3 KO mice compared with WT mice. This is consistent with findings in IL‐4 KO mice, which display a significantly worsened disease course, but where the cellular profile of the inflammatory infiltrate was not altered, nor was lesion density.^18^ However, our data suggest that the nature of the infiltrate is changed, as we observed a marked reduction in the expression of the critical type 1 cytokine IFN‐γ in the spinal cord. Moreover, although expression of the cytokine IL‐17a is reduced in the spinal cord, it is increased in the lymph nodes of Tyro3 KO mice compared with WT mice. This is an important observation because although Tyro3 KO mice have an ameliorated disease severity, incidence of disease was similar to WT mice, and there was a trend toward slightly earlier onset of EAE in the absence of Tyro3. Unlike IL‐4, which is not required for EAE induction,^18^ IL‐17a has been demonstrated to be critical for the priming but not for the effector function of Th17 cells in EAE.^19^ Furthermore, it has previously been demonstrated that GAS6 signaling reduces expression of IL‐17a in people with concurrent MS and helminth infection,^41^ suggesting that the increased expression of IL‐17a in Tyro3 KO mice is a result of the loss of the Gas6‐Tyro3 signaling axis in these mice. Given the reduction in disease severity, it was unsurprising that levels of both IL‐17A and IFN‐γ were reduced in the spinal cord. The additional reduction in IL‐4 was more surprising. Given that the levels of infiltrate were unchanged between Tyro3 WT and KO mice, it is possible that there is a general inhibition of T cell responses in the KO mice that may be contributing to the decreased disease severity in these animals. An alternative explanation may be that while the levels of infiltrate are similar, there are differences between Tyro3 KO and WT mice in expression of IL‐4 which balance the differences in infiltrate IL‐4. A deeper cell‐specific analysis of cytokine expression in the spinal cord would be required to differentiate between these possibilities.

Our findings raise important questions in respect to the potential benefits and risks of Gas6‐based therapies to promote remyelination in MS. On the one hand, there is clear evidence that administration of Gas6 directly to the CNS promotes remyelination and functional recovery in pre‐clinical models of MS.^6^, ^9^, ^15^ On the other hand, we have now demonstrated that global loss of Gas6 or Tyro3 reduces the severity of disease following induction of EAE, likely through alteration in T cell responses. Adding further complexity is our finding that although disease severity is ameliorated in the absence of Gas6 and Tyro3, susceptibility is not reduced and IL‐17a expression is increased. Given that IL‐17a is generally considered to have a pathological role in the context of MS,^42^ the increase in lymph node IL‐17a in Tyro3 KO mice would suggest that, despite the amelioration of disease severity in these mice, a therapy aimed at reducing Tyro3 activation is not likely to have clear‐cut positive outcomes. Careful dissection of the cell‐specific roles of the TAM receptors using conditional knock out mouse lines, potentially in combination with pharmacological enhancement of TAM signaling with selective agonists, will likely be necessary to fully elucidate the roles of the TAM receptors during inflammatory demyelination.

## METHODS

### Materials

Unless otherwise stated, all reagents were obtained from Sigma‐Aldrich (St. Louis, MO, USA).

### Animal resources and ethics

C57Bl/6J mice were obtained from the Animal Resources Centre (ARC, Perth, WA, Australia). Mice in which Tyro3 has been deleted (Tyro3 KO) were originally a gift from Professor Greg Lemke (Salk Institute of Biological Studies, La Jolla, CA, USA).^43^ Mice in which Gas6 has been deleted (Gas6 KO) were originally obtained from Professor Peter Carmeliet (The Center for Transgene Technology and Gene Therapy, Flanders Interuniversity Institute for Biotechnology, Leuven, Belgium^44^). Mice (Tyro3 KO and Gas6 KO) were fully backcrossed (> 10 generations) onto the C57Bl/6 background prior to experimental studies. Experimental cohorts were derived from heterozygous matings and used equal numbers of male and female mice; all experiments utilized littermate wild‐type (WT) mice as controls. All mice were maintained in a specific pathogen‐free environment during breeding and experiments. All animal experiments were approved by the Florey Institutional Animal Ethics Committee and performed according to National Health and Medical Research Council Australia guidelines.

### Induction of experimental autoimmune encephalomyelitis

Experimental autoimmune encephalomyelitis was induced in mice using the MOG_35–55_ peptide [MEVGWYRSPFSRVVHLYRNGK (Mimotopes, Melbourne, VIC, Australia)] at a concentration of 1 mg mL^−1^ emulsified in an equal volume of Freund's complete adjuvant containing 5 mg mL^−1^ of *M. tuberculosis* (Becton Dickinson, Franklin Lakes, NJ, USA). Mice were immunized via subcutaneous injection of emulsified peptide into both flanks and the base of the tail. Mice also received intraperitoneal injections of pertussis toxin (400 ng/injection; Sapphire Bioscience Pty Ltd, Redfern, NSW, Australia) on the day of induction and 3 days later. Mice were assessed each day for clinical symptoms following the day of induction and graded using the following scale: 0, healthy; 1.0, tail weakness; 1.5, tail weakness and hindlimb weakness; 2.0, complete tail paralysis; 2.25, complete tail paralysis and some hindlimb weakness; 2.5, complete tail paralysis and severe hindlimb weakness; 2.75, complete tail paralysis and paralysis of one hindlimb; 3.0, complete tail and hindlimb paralysis; 3.5, complete tail and hindlimb paralysis with forelimb weakness and loss of righting reflex; 4.0, death. Disease incidence was calculated as the percentage of mice that reached a score ≥ 1 within an experimental group.

### Collection of mouse tissue and purification of cell populations

Mice were killed by injection of sodium pentobarbitone [*i.p*., 100 mg/kg (Carros, France)] in mouse tonicity (MT)‐PBS (16.3 mM Na_2_HPO_4_, 62.7 mM_NaH2PO4_, 148 mM NaCl in MilliQ H_2_O). Spleens, spinal cords, and inguinal lymph nodes were removed by dissection. Spinal cords and inguinal lymph nodes were snap frozen in liquid nitrogen and stored at −80° prior to processing for RNA isolation. Spleens were immediately processed for cell separation as described below. It has been demonstrated that spleens and lymph nodes are similar in baseline proportions of immune cells.^45^

In order to obtain single‐cell suspensions, spleens were gently ground using the plunger of a 3 mL syringe through a 40‐μm filter, using FACS buffer (2 mM EDTA, 2% FBS [Scientifix, Clayton, Victoria, Australia] in 1×PBS) to wash cells through the filter. After obtaining single‐cell suspensions, cells were pelleted by centrifugation at 300 *g* for 10 min. To lyse red blood cells, cell suspensions were resuspended in 5 mL of red blood cell lysis buffer (154.4 mM NH_4_Cl, 10 mM KHCO_3_, 1 mM EDTA in MilliQ H_2_O) and incubated for 7 min at room temperature. To stop lysis, 5 mL of PBS was added to the suspension, and cells were pelleted again by centrifugation at 300 *g* for 10 min and resuspended in FACS buffer. After counting, cells were resuspended in 90 μL FACS buffer with 10 μL of mouse Fc receptor blocker (Miltenyi Biotec, Bergisch Gladbach, Germany) per 10^7^ cells and incubated at 4°C for 10 min. Cells were then washed using FACS buffer, pelleted, and resuspended in a suitable volume for cell separation by MACS or FACS.

Lymphocytes were purified from total splenocytes using MACS. Splenocytes were labeled with magnetic microbeads (Miltenyi Biotec) specific for either mouse CD4, CD8, or CD19 by resuspending in 90 μL FACS buffer with 10 μL of the appropriate antibody per 10^7^ cells and incubating at 4°C for 15 min. After washing with FACS buffer, splenocytes were resuspended with 500 μL FACS buffer per 10^8^ cells. Positive selection of cells was then conducted using LS columns (Miltenyi Biotec) on an autoMACS Pro Separator (Miltenyi Biotec) using the standard positive selection “Possel” program.

Tissue macrophages and dendritic cells were purified from total splenocytes using FACS. Splenocytes were incubated at 4°C for 15 min with a custom negative‐selection, depletion solution based on depletion solutions from Miltenyi Biotec (Catolog #130‐091‐262 and 130‐091‐169), consisting of magnetic microbeads (Miltenyi Biotec) specific for CD4 (Th cells), CD19 (B cells), Ter119 (erythrocytes), CD45R (B cells), and CD49b (NK cells) at a concentration of 1:9 per microbead. Following washing, unwanted cells were depleted by passing the cell suspension through a magnetic LS column (Miltenyi Biotec) and collecting the flow‐through. Next, the depleted flow‐through splenocytes were labeled for positive selection with fluorophore‐conjugated antibodies specific for both mouse CD11b and CD11c (Miltenyi Biotec) by resuspending splenocytes in FACS buffer containing CD11b‐PE (1:200) and CD11c‐FITC (1:100) and incubating at 4°C for 30 min. Splenocytes were then resuspended in FACS buffer and sorted at the Melbourne Brain Centre Flow Cytometry Facility on a FACSAriaTM III (BD Bioscience, California, USA). For macrophages, all CD11b single positive (CD11b^+^CD11c^−^) cells were isolated. For DCs, all CD11c^+^ and CD11c^+^CD11b^+^ cells were isolated.

### Assessment of cell density and lesion volume

Mice were anesthetized by intra‐peritoneal injection of sodium pentobarbitone (100 mg/kg, Virbac) in MT‐PBS and then perfused intracardially with 20 mL MT‐PBS at 37°C followed by 20 mL of ice cold 4% (w/v) paraformaldehyde (Merck KGaA, Darmstadt, Germany). Spinal cords were dissected from the vertebral column and the lumbar expansion was excised. The excised spinal cord was bisected at L3. The lower (L3–L5) was post‐fixed in 4% PFA for 48 h, dehydrated through graded alcohols. Tissue was embedded in paraffin blocks and 5‐μm axial sections were cut and stained with H&E (Melbourne Histology Platform, VIC, Australia). Every sixth section (total four per mouse) was imaged using an Olympus IX81 microscope. Whole spinal cord area and lesion area (identified as regions of dense hematoxylin‐stained nuclei) were measured using ImageJ version 1.52a (National Institutes of Health, Bethesda, MD, USA) and the lesion burden (lesion area/total area) was calculated. The upper (L1–3) lumbar region was post‐fixed for 30 min in ice cold 4% PFA and then stored overnight at 4°C in 30% sucrose (Amresco, Solon, OH, USA) in MT‐PBS for cryoprotection. Tissue was then embedded in Tissue‐Tek optimum cutting temperature compound (Sakura Fintech, Tokyo, Japan) and frozen on dry ice. For lower lumbar expansions, axial sections were cut using a cryostat at 10 μm intervals and collected onto Superfrost plus slides (Menzel Glass, Braunschweig, Germany). To assess cell density, tissue sections were incubated with anti‐IBA1 antibody (1:1000, FUJIFILM Wako, Richmond, VA, USA, 019‐19741). Positive cells were visualized with an appropriate secondary antibody conjugated to a fluorescent tag. Following immunohistochemical staining, sections were imaged using an Axioplan microscope (Carl Zeiss, Oberkochen, Germany). The area of the dorsal column or rostral corpus callosum was calculated using ImageJ version 1.52a and all results expressed as cells/mm^2^. All counts were performed by a researcher blind to animal sex and genotype.

### Assessment of gene expression

RNA was isolated from purified cells or whole tissue (whole spinal cord or whole inguinal lymph nodes) using the RNeasy Plus minikit (Qiagen, Venlo, Netherlands). cDNA was generated using the Taqman reverse transcription kit (Thermofisher Scientific, Waltham, MA, USA). Quantitative PCR was performed on a ViiA7 instrument (Thermofisher Scientific) using SYBR green master mix (Thermofisher Scientific) according to the manufacturer's instructions. Gene expression levels were determined using the comparative Ct method.^46^ 18S was used as the internal reference gene, and then the data were normalized to an unchallenged mouse and expressed as log_2_. A list of primers used is given in Supplementary Table 1.

### Statistical analyses

Statistical analysis was performed using PRISM software (GraphPad, v.10, Boston, MA, USA). Gene expression following the induction of EAE was analyzed using one‐way ANOVA followed by Dunnett's multiple comparison *post‐hoc* test, with each timepoint compared to unchallenged. Mean differences in the clinical course of EAE were assessed using the Mann–Whitney *U*‐test. Incidence of EAE was assessed using the Gehan–Breslow–Wilcoxon method. The Student's *t*‐tests were used to compare gene expression and lesion or cell density at day 16 post‐EAE induction.

## AUTHOR CONTRIBUTIONS

**Michele D Binder:** Conceptualization; data curation; formal analysis; funding acquisition; investigation; methodology; project administration; resources; supervision; visualization; writing – original draft; writing – review and editing. **Mohammad Asadian:** Data curation; investigation. **Darnell Leepel:** Data curation; investigation. **Gerry ZM Ma:** Data curation; investigation. **Andrea Aprico:** Data curation; investigation; writing – review and editing. **Liz Barreto‐Arce:** Data curation; investigation; writing – review and editing. **Trevor J Kilpatrick:** Conceptualization; funding acquisition; project administration; supervision; writing – review and editing. **Sarrabeth Stone:** Conceptualization; funding acquisition; investigation; methodology; project administration; supervision; writing – review and editing.

## CONFLICT OF INTEREST

This project was partially funded by Novartis Pharmaceuticals Australia. We declare no other conflict of interest.

## Supporting information


Supplementary table 1

## Data Availability

The data that support the findings of this study are available from the corresponding author upon reasonable request.

## References

[1] Tremlett H , Zhao Y , Devonshire V , UBC Neurologists . Natural history comparisons of primary and secondary progressive multiple sclerosis reveals differences and similarities. J Neurol 2009; 256: 374–381. 19308306 10.1007/s00415-009-0039-7

[2] Lee Y , Morrison BM , Li Y , *et al*. Oligodendroglia metabolically support axons and contribute to neurodegeneration. Nature 2012; 487: 443–448. 22801498 10.1038/nature11314 PMC3408792

[3] Binder MD , Cate HS , Prieto AL , *et al*. Gas6 deficiency increases oligodendrocyte loss and microglial activation in response to cuprizone‐induced demyelination. J Neurosci 2008; 28: 5195–5206. 18480276 10.1523/JNEUROSCI.1180-08.2008 PMC3844801

[4] Binder MD , Xiao J , Kemper D , Ma GZM , Murray SS , Kilpatrick TJ . Gas6 increases myelination by oligodendrocytes and its deficiency delays recovery following cuprizone‐induced demyelination. PLoS One 2011; 6: e17727. 21423702 10.1371/journal.pone.0017727 PMC3053381

[5] Gruber RC , Ray AK , Johndrow CT , *et al*. Targeted GAS6 delivery to the CNS protects axons from damage during experimental autoimmune encephalomyelitis. J Neurosci 2014; 34: 16320–16335. 25471571 10.1523/JNEUROSCI.2449-14.2014 PMC4252545

[6] Tsiperson V , Li X , Schwartz GJ , Raine CS , Shafit‐Zagardo B . GAS6 enhances repair following cuprizone‐induced demyelination. PLoS One 2010; 5: e15748. 21203420 10.1371/journal.pone.0015748 PMC3009745

[7] Binder MD , Kilpatrick TJ . TAM receptor signalling and demyelination. Neurosignals 2009; 17: 277–287. 19816064 10.1159/000231894

[8] Lew ED , Oh J , Burrola PG , *et al*. Differential TAM receptor‐ligand‐phospholipid interactions delimit differential TAM bioactivities. eLife 2014; 3: e03385. 25265470 10.7554/eLife.03385 PMC4206827

[9] Asadian N , Aprico A , Chen M , *et al*. The therapeutic effect of GAS6 in remyelination is dependent upon Tyro3. Glia 2024; 72: 1392–1401. 38572807 10.1002/glia.24534

[10] Binder MD , Fox AD , Merlo D , *et al*. Common and low frequency variants in MERTK are independently associated with multiple sclerosis susceptibility with discordant association dependent upon HLA‐DRB1*15:01 status. PLoS Genet 2016; 12: e1005853. 26990204 10.1371/journal.pgen.1005853 PMC4798184

[11] Rothlin CV , Ghosh S , Zuniga EI , Oldstone MBA , Lemke G . TAM receptors are pleiotropic inhibitors of the innate immune response. Cell 2007; 131: 1124–1136. 18083102 10.1016/j.cell.2007.10.034

[12] Lu Q , Lemke G . Homeostatic regulation of the immune system by receptor tyrosine kinases of the tyro 3 family. Science 2001; 293: 306–311. 11452127 10.1126/science.1061663

[13] Cabezón R , Carrera‐Silva EA , Flórez‐Grau G , *et al*. MERTK as negative regulator of human T cell activation. J Leukoc Biol 2015; 97: 751–760. 25624460 10.1189/jlb.3A0714-334R PMC4370049

[14] Weinger JG , Brosnan CF , Loudig O , *et al*. Loss of the receptor tyrosine kinase Axl leads to enhanced inflammation in the CNS and delayed removal of myelin debris during experimental autoimmune encephalomyelitis. J Neuroinflammation 2010; 8: 49. 10.1186/1742-2094-8-49 PMC3121615 21569627

[15] Gruber RC , Ray AK , Johndrow CT , *et al*. Targeted GAS6 delivery to the CNS protects axons from damage during experimental autoimmune encephalomyelitis. J Neurosci 2014; 34: 16320–16335. 25471571 10.1523/JNEUROSCI.2449-14.2014 PMC4252545

[16] Blades F , Aprico A , Akkermann R , Ellis S , Binder MD , Kilpatrick TJ . The TAM receptor TYRO3 is a critical regulator of myelin thickness in the central nervous system. Glia 2018; 66: 2209–2220. 30208252 10.1002/glia.23481

[17] Naves R , Singh SP , Cashman KS , *et al*. The interdependent, overlapping, and differential roles of type I and II IFNs in the pathogenesis of experimental autoimmune encephalomyelitis. J Immunol 2013; 191: 2967–2977. 23960239 10.4049/jimmunol.1300419 PMC3779698

[18] Falcone M , Rajan AJ , Bloom BR , Brosnan CF . A critical role for IL‐4 in regulating disease severity in experimental allergic encephalomyelitis as demonstrated in IL‐4‐deficient C57BL/6 mice and BALB/c mice. J Immunol 1998; 160: 4822–4830. 9590229

[19] McGinley AM , Sutton CE , Edwards SC , *et al*. Interleukin‐17A serves a priming role in autoimmunity by recruiting IL‐1β‐producing myeloid cells that promote pathogenic T cells. Immunity 2020; 52: 342–356. 32023490 10.1016/j.immuni.2020.01.002

[20] Nguyen LT , Aprico A , Nwoke E , *et al*. Mertk‐expressing microglia influence oligodendrogenesis and myelin modelling in the CNS. J Neuroinflammation 2023; 20: 253. 37926818 10.1186/s12974-023-02921-8 PMC10626688

[21] Hoehn HJ , Kress Y , Sohn A , Brosnan CF , Bourdon S , Shafit‐Zagardo B . Axl^−/−^ mice have delayed recovery and prolonged axonal damage following cuprizone toxicity. Brain Res 2008; 1240: 1–11. 18804096 10.1016/j.brainres.2008.08.076

[22] Ray AK , DuBois JC , Gruber RC , *et al*. Loss of Gas6 and Axl signaling results in extensive axonal damage, motor deficits, prolonged neuroinflammation, and less remyelination following cuprizone exposure. Glia 2017; 65: 2051–2069. 28925029 10.1002/glia.23214 PMC5643251

[23] Silva EAC , Chan PY , Joannas L , *et al*. T cell‐derived protein S engages TAM receptor signaling in dendritic cells to control the magnitude of the immune response. Immunity 2013; 39: 160–170. 23850380 10.1016/j.immuni.2013.06.010 PMC4017237

[24] Kivisäkk P , Imitola J , Rasmussen S , *et al*. Localizing central nervous system immune surveillance: meningeal antigen‐presenting cells activate T cells during experimental autoimmune encephalomyelitis. Ann Neurol 2009; 65: 457–469. 18496841 10.1002/ana.21379 PMC3305810

[25] Tirado‐Gonzalez I , Descot A , Soetopo D , *et al*. AXL inhibition in macrophages stimulates host‐versus‐leukemia immunity and eradicates naive and treatment resistant leukemia. Cancer Discov 2021; 11: 2924–2943. 34103328 10.1158/2159-8290.CD-20-1378 PMC7611942

[26] Rigoni TS , Vellozo NS , Guimarães‐Pinto K , *et al*. Axl receptor induces efferocytosis, dampens M1 macrophage responses and promotes heart pathology in *Trypanosoma cruzi* infection. Commun Biol 2022; 5: 1421. 36581764 10.1038/s42003-022-04401-w PMC9800583

[27] D'Onghia D , Colangelo D , Bellan M , *et al*. Gas6/TAM system as potential biomarker for multiple sclerosis prognosis. Front Immunol 2024; 15: 1362960. 38745659 10.3389/fimmu.2024.1362960 PMC11091300

[28] Ma GZ , Giuffrida LL , Gresle MM , *et al*. Association of plasma levels of protein S with disease severity in multiple sclerosis. Mult Scler J 2015; 1: 2055217315596532. 10.1177/2055217315596532 PMC5433335 28607700

[29] Rosenstein I , Novakova L , Kvartsberg H , *et al*. Tyro3 and Gas6 are associated with white matter and myelin integrity in multiple sclerosis. J Neuroinflammation 2024; 21: 320. 39673059 10.1186/s12974-024-03315-0 PMC11645787

[30] Weinger JG , Omari KM , Marsden K , Raine CS , Shafit‐Zagardo B . Up‐regulation of soluble Axl and Mer receptor tyrosine kinases negatively correlates with Gas6 in established multiple sclerosis lesions. Am J Pathol 2009; 175: 283–293. 19541935 10.2353/ajpath.2009.080807 PMC2708814

[31] Goudarzi S , Rivera A , Butt AM , Hafizi S . Gas6 promotes oligodendrogenesis and myelination in the adult central nervous system and after lysolecithin‐induced demyelination. ASN Neuro 2016; 8: 1759091416668430. 27630207 10.1177/1759091416668430 PMC5027908

[32] Shaw MK , Lorens JB , Dhawan A , *et al*. Local delivery of interleukin 4 by retrovirus‐transduced T lymphocytes ameliorates experimental autoimmune encephalomyelitis. J Exp Med 1997; 185: 1711–1714. 9151908 10.1084/jem.185.9.1711 PMC2196296

[33] Falcone M , Bloom BR . A T helper cell 2 (Th2) immune response against non‐self antigens modifies the cytokine profile of autoimmune T cells and protects against experimental allergic encephalomyelitis. J Exp Med 1997; 185: 901–908. 9120396 10.1084/jem.185.5.901 PMC2196156

[34] Allen JE , Sutherland TE . Host protective roles of type 2 immunity: parasite killing and tissue repair, flip sides of the same coin. Semin Immunol 2014; 26: 329–340. 25028340 10.1016/j.smim.2014.06.003 PMC4179909

[35] Molofsky AB , Locksley RM . The ins and outs of innate and adaptive type 2 immunity. Immunity 2023; 56: 704–722. 37044061 10.1016/j.immuni.2023.03.014 PMC10120575

[36] Chan PY , Silva EAC , Kouchkovsky DD , *et al*. The TAM family receptor tyrosine kinase TYRO3 is a negative regulator of type 2 immunity. Science 2016; 352: 99–103. 27034374 10.1126/science.aaf1358 PMC4935984

[37] Chakma CR , Good‐Jacobson KL . Requirements of IL‐4 during the generation of B cell memory. J Immunol 2023; 210: 1853–1860. 37276051 10.4049/jimmunol.2200922

[38] King C , Hoenger RM , Cleary MM , *et al*. Interleukin‐4 acts at the locus of the antigen‐presenting dendritic cell to counter‐regulate cytotoxic CD8^+^ T‐cell responses. Nat Med 2001; 7: 206–214. 11175852 10.1038/84659

[39] Sun D , Whitaker JN , Huang Z , *et al*. Myelin antigen‐specific CD8^+^ T cells are Encephalitogenic and produce severe disease in C57BL/6 mice. J Immunol 2001; 166: 7579–7587. 11390514 10.4049/jimmunol.166.12.7579

[40] Lyons J , Ramsbottom MJ , Cross AH . Critical role of antigen‐specific antibody in experimental autoimmune encephalomyelitis induced by recombinant myelin oligodendrocyte glycoprotein. Eur J Immunol 2002; 32: 1905–1913. 12115610 10.1002/1521-4141(200207)32:7<1905::AID-IMMU1905>3.0.CO;2-L

[41] Wilczyñski JMO , Olexen CM , Errasti AE , *et al*. GAS6 signaling tempers Th17 development in patients with multiple sclerosis and helminth infection. PLoS Pathog 2020; 16: e1009176. 33347509 10.1371/journal.ppat.1009176 PMC7785232

[42] Mills KHG . IL‐17 and IL‐17‐producing cells in protection versus pathology. Nat Rev Immunol 2023; 23: 38–54. 35790881 10.1038/s41577-022-00746-9 PMC9255545

[43] Lu Q , Gore M , Zhang Q , *et al*. Tyro‐3 family receptors are essential regulators of mammalian spermatogenesis. Nature 1999; 398: 723–728. 10227296 10.1038/19554

[44] Angelillo‐Scherrer A , de Frutos PG , Aparicio C , *et al*. Deficiency or inhibition of Gas6 causes platelet dysfunction and protects mice against thrombosis. Nat Med 2001; 7: 215–221. 11175853 10.1038/84667

[45] Li H , Wang D , Zhou X , *et al*. Characterization of spleen and lymph node cell types via CITE‐seq and machine learning methods. Front Mol Neurosci 2022; 15: 1033159. 36311013 10.3389/fnmol.2022.1033159 PMC9608858

[46] Livak KJ , Schmittgen TD . Analysis of relative gene expression data using real‐time quantitative PCR and the 2−ΔΔ CT Method. Methods 2000; 25: 402–408. 10.1006/meth.2001.1262 11846609

