# Supplementary material for: Tyro3 deletion is protective in experimental autoimmune encephalomyelitis
Source: Immunol Cell Biol. 2025 Aug 8;103(9):857–70. doi: 10.1111/imcb.70054 (PMC12521954; doi:10.1111/imcb.70054)
Supplement: Supplementary file 1 — Supplementary table 1 [file IMCB-103-857-s001.pdf]

Supplementary table 1: Sequences of primers used in quantitative PCR

Gene	Primers (5'-3')
18S	Forward: CGAACGTCTGCCCTATCAACTT<br>Reverse: ACCCGTGGTCACCATGGTA
Tyro3	Forward: TGGCTGAGCTGCTCCTACTTTA<br>Reverse: TGGGCAGTGCTGAGTTCCA
Axl	Forward: TCTAATGTTGGCCTGATCTTCGT<br>Reverse: GCCTCTTGTGACTTAGATGCTGAG
Mertk	Forward: GACGGTGTCCAAGGGTGTACAT<br>Reverse: CTGTACTGTTGAGGATATGGACTTCAG
Gas6	Forward: GCTGCAGCTTCGGTACAATG<br>Reverse: ACATGCCGTGGTTGATGGTT
Pros1	Forward: TTTGATGATAGTGCATTGCAAGTG<br>Reverse: CGTCTGATGCACCCCTGAA
IL-4	Forward: CAGCAACGAAGAACACCACA<br>Reverse: ACCTTGGAAGCCCTACAGAC
IL-17A	Forward: GAAGGCCCTCAGACTACCTC<br>Reverse: TCTTCATTGCGGTGGAGAGT
1L-10	Forward: ATGGGAGGGGTTCTTCCTTG<br>Reverse: AGTGTGGCCAGCCTTAGAAT
1L-5	Forward: ACATGCTGGGCCTTACTTCT<br>Reverse: GTAAACTGGGGGAGGCTTCT
IL-13	Forward: ACAAAGCAACTGTTTCGCCA<br>Reverse: CTCTCCCCAGCAAAGTCTGA
IFN-γ	Forward: AAAATCCTGCAGAGCCAGAT<br>Reverse: CATGAATGCATCCTTTTTCG
